# Poly-*N*-isopropylacrylamide (pNIPAM): a reversible bioadhesive for sclerotomy closure

**DOI:** 10.1186/s40942-016-0048-5

**Published:** 2016-10-03

**Authors:** Luiz H. Lima, Yael Morales, Thiago Cabral

**Affiliations:** 1 Doheny Eye Institute, University of California Los Angeles (UCLA), Los Angeles, CA USA; 2 Federal University of Sao Paulo (UNIFESP), Rua Botucatu, 821, Vila Clementino, Sao Paulo, São Paulo 04023-062 Brazil

**Keywords:** Pars plana vitrectomy, pNIPAM, Retina, Thermosensitive glue

## Abstract

**Purpose:**

To determine the safety and efficacy of poly-*N*-isopropylacrylamide, a thermoresponsive adhesive, for sutureless sclerotomy closure in rabbits.

**Methods:**

Eight rabbits were randomized into three groups: short-term acute, mid-term chronic, and long-term chronic studies. A corresponding control group in which the scleral wounds were sutured by 6–0 vicryl sutures was assigned for each study group. A 20-gauge sclerotomy was performed following a core vitrectomy and 0.1 mL of 50 % liquid poly-*N*-isopropylacrylamide was applied to the scleral wounds. Before the polymer application, the scleral surface was raised above 32 °C using a halogen bulb lamp. Follow-up exams included color external and fundus photography, fundus fluorescein angiography, optical coherence tomography, and electroretinography. After the last follow-up assessment, the rabbits were sacrificed and histopathological studies on the scleral incision sites were performed.

**Results:**

Scleral wound healing was observed in the long-term chronic study rabbits. Histological studies were able to identify poly-*N*-isopropylacrylamide polymer at the sclerotomy site in the mid-term chronic study rabbits. Besides iatrogenic cataracts due to mechanical instrument touch in 2 rabbits, no other ocular abnormalities were identified in any of the eyes in the perioperative setting or during the follow-up period. Cornea, retina, and vitreous remained unaffected, and no abnormal inflammatory reaction or endophthalmitis was noticed in the 3 study groups. Filtering blebs indicative of leakage through the sclerotomies were not observed during the follow-up period.

**Conclusion:**

Poly-*N*-isopropylacrylamide may provide effective in vitro scleral adhesion above 32 °C. Clinical studies are required to evaluate its utility in patients undergoing pars plana vitrectomy.

## Background

Even with the advent of smaller gauge surgery, a number of pars plana vitrectomies continue to be performed using 20 gauge incisions that require sutures for closure [1–3]. The sutures that are commonly used to close the sclerotomies can induce granulomas, inflammation, irritation, and astigmatism. Moreover, some smaller gauge sutureless incisions may not be water-tight, leading to hypotony and an increased incidence of endophthalmitis. Bioadhesives such as cyanoacrylate and fibrin glue have been used to close leaking sclerotomies after vitrectomy surgery. However, the use of these compounds resulted in complications such as incomplete sclerotomy closure, ocular hypotony and surface roughness leading to ocular irritation [4–6].

The use of thermally responsive polymers in biomedical applications has been widely investigated. Thermally responsive polymers exhibit a lower critical solution temperature (LCST), below which the polymers are soluble. When the temperature is raised above the LCST, these polymers first undergo a phase transition; they then collapse and form aggregates. This phenomenon is reversible such that when the temperature is lowered, the polymers once again become soluble [7].

Poly-*N*-isopropylacrylamide (pNIPAM) is a thermo-responsive polymer with inverse solubility and a reversible phase transition upon heating [8]. It has an LCST value of approximately 32 °C. Below 32 °C, it is hydrophilic and water soluble; above 32 °C, it is hydrophobic and becomes a viscous gel that is strongly adherent to tissue. pNIPAM is soluble at room temperature, but it phase separates at body temperature (37 °C); it therefore can be used as a linear polymer, a hydrogel, or a copolymer [9–12]. For example, pNIPAM has been used for drug targeting in solid tumors with local hyperthermia and in thermosensitive coatings or micelles for controlled release of the drug. In addition, pNIPAM polymers have been used in eye drop preparations and as a new embolic material in neurosurgery [13–15].

Although pNIPAM has been studied as a new ocular drug delivery platform to the posterior segment of the eye and as an adhesive for retinal implants [16–20] there is no report regarding its application for scleral wound healing. The purpose of the present feasibility study was to evaluate the safety and efficacy of pNIPAM as an adhesive for the closure of sclerotomies.

## Methods

### pNIPAM synthesis

We synthesized pNIPAM through bulk polymerization of 1.0 g of *N*-isopropylacrylamide with 0.0145 g of 2,2-azobisisobutyronitrile in 14 mL of ethanol. The mixture was heated in an oil bath set at 65 °C for 16 h under constant stirring. After the ethanol was removed under vacuum, the polymer was dissolved in a minimal amount of tetrahydrofuran and recrystalized by the addition of ether. The solid precipitates were collected and air dried. The solid pNIPAM was then dissolved in phosphate-buffered saline at different concentrations (20, 30, and 50 %).

### pNIPAM in vivo studies

**Fig. 1:**
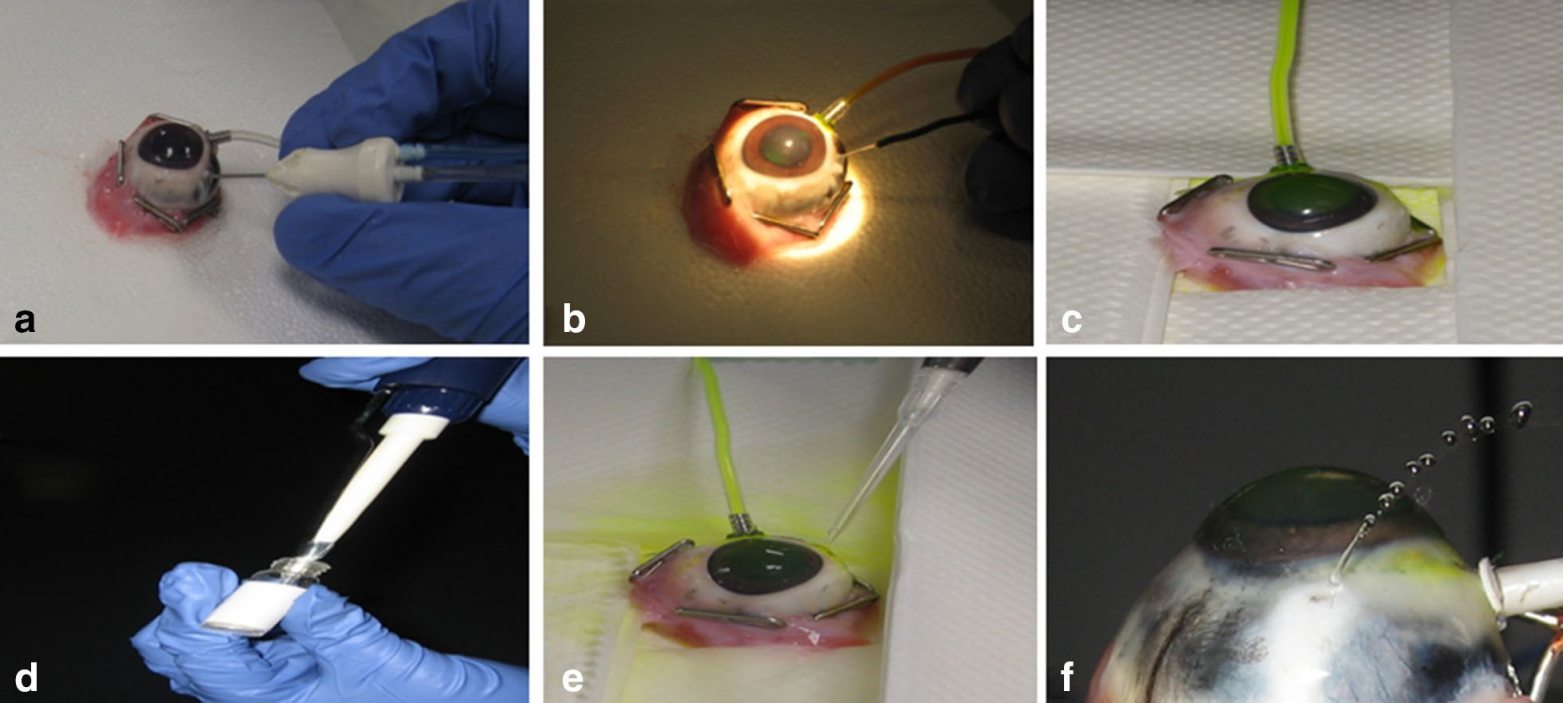
**a**–**f** Sequence of events in the porcine eye experiment. **a** Core vitrectomy before patency of balanced salted solution (BSS) flow through the sclerotomy. **b** The thermocouple measuring the sclera temperature to confirm levels higher than 31 °C. **c** Fluorescein dye injection in the infusion line before the adhesive placement for a better visualization of the BSS flow through the sclerotomy. **d** Removal of 50 % liquid poly-*N*-isopropylacrylamide (PNIPAM). **e** PNIPAM application on the 20-gauge sclerotomy site. **f** BSS leakage through the sclerotomy wound at 80 mmHg bottle height

In preliminary studies, we examined the use of pNIPAM for sclerotomy closure in 10 enucleated porcine eyes (Sierra for Medical Science, Whittier, CA, USA). Following a conjunctiva sectioning, a two-port pars plana vitrectomy was performed in the porcine eye with a 20-gauge system (Bausch & Lomb, Rochester, NY USA) using perpendicular scleral incisions. A 20-gauge MVR blade was used for the 2 sclerotomies (one for the infusion line and the other for the vitreous cutter). We performed a core vitrectomy using a Millenium Microsurgical System (Bausch & Lomb, Rochester, New York, USA) and the patency of the 20-gauge sclerotomy was confirmed by visualizing room temperature balanced salt solution (BSS, Baxter, Deerfield, Illinois, USA) flow through the sclerotomy. Then, the scleral surface was dried with cotton-tips, and the scleral temperature was raised above 32 °C using a halogen bulb lamp. The scleral temperature was monitored with a thermocoupled 24-gauge hypodermic probe (Omega Engineering, Inc., Stamford, CT, USA) connected to a tele-thermometer (model DP 460-T Omega Engineering, Inc., Stamford, CT, USA) and placed on the scleral surface immediately adjacent to the applied glue. An amount of 0.1 mL of liquid pNIPAM was applied at various concentrations (20, 30, and 50 %) to the sclerotomy wounds. After allowing the pNIPAM to dry for a few minutes, we turned on the infusion fluid which contained fluorescein dye. The bottle with BSS was raised until leakage was noted through the scleral wound (Fig. 1). The temperature was monitored and kept above 32 °C during the entire experiment. In the control group, seven scleral wounds were left untreated and six wounds were closed with a traditional 3-pass running configuration by 6–0 vicryl suture. Before the sclerotomy closure, these porcine eyes underwent the same surgical protocol used for the study group.

Animals were used in accordance with the ARVO statement on the Use of Animals in Ophthalmic and Visual Research. After Institutional Animal Care and Use committee and institutional review board approval from the University of California Los Angeles (UCLA), a total of 16 animals were used in the present study. Eight New Zealand pigmented rabbits weighing between 2 and 4 kg were randomized into three groups for the short-term acute (2 h), mid-term chronic (14 and 30 days), and long-term chronic (6 months) studies. Eight animals were used for controls as described below.

In the short-term acute study, pNIPAM was applied to the 20-gauge sclerotomies in four rabbit eyes for 2 h and the sclerotomy was monitored while the bottle height was kept constant at 70 mmHg. Scleral temperature was maintained above 32 °C in 2 rabbits and was allowed to decrease without heating to 20 °C in the other 2 rabbits. The four rabbits in the short-term acute study were euthanized by intracardiac injection of pentobarbital after 2 h of observation. In the mid-term chronic study, pNIPAM was applied to the sclerotomy in two rabbits; one of these rabbits was followed up for 14 days and the second for 30 days. In the long-term chronic study, pNIPAM was applied to 2 rabbits which were followed up for 6 months. In all study rabbits, pNIPAM was applied in a concentration of 50 %.

A two-port pars plana vitrectomy was performed in the rabbit eye with a 20-gauge system (Bausch & Lomb, Rochester, NY USA) using perpendicular scleral incisions after conjunctiva sectioning. The 20-gauge sclerotomy procedure was performed using a MVR blade and under sterile conditions using an operating microscope. A speculum was used to keep the eyelids open. Two sclerotomies were performed at a distance of 180 degree from each other, one was for the infusion cannula and the other for the vitreous cutter. A core vitrectomy was performed using a Millenium Microsurgical System (Bausch & Lomb, Rochester, New York, USA). Following the documenting of the patency of the sclerotomy as evidenced by efflux of BSS from the sclerotomy, the scleral surface was dried with cotton-tips before the polymer application and the scleral temperature was raised above 32 °C using a halogen bulb lamp. Liquid pNIPAM was applied with a 1 mL syringe to the temporal quadrant sclerotomy in the same amount (0.1 mL) and concentration (50 %) in all rabbits. The study animals were carefully examined by trained veterinarians during the surgical procedure and in the postoperative follow-up period. For each of the 3 study groups, there was a corresponding control group with equal number of rabbits that underwent identical surgical protocol before the sclerotomy closure. In this control group, the scleral wounds were sutured by 6–0 vicryl sutures using a traditional 3-pass running configuration and conjunctival wounds by 8–0 vicryl sutures.

At regular intervals, the rabbits underwent routine evaluations, including slit-lamp examination, intraocular pressure measurement, indirect ophthalmoscopy, external and fundus color photography, fundus fluorescein angiography (FA), optical coherence tomography (OCT) and electroretinography (ERG). Slit- lamp examinations and indirect ophthalmoscopy were performed preoperatively, immediately after injection and on days 1, 2, 4, 7, and 14, and monthly thereafter for 6 months. Intraocular pressure was measured with a tonometer at each examination. OCT, FA, and external and fundus photography were performed preoperatively, on day 14 and then monthly for 6 months. ERGs were recorded preoperatively, on day 14 (one rabbit), and monthly thereafter, using an Espion Visual Electrophysiology unit (Diagnosys LLC, Lowell, MA, USA) [21, 22].

After the last follow-up examination, the rabbits were sacrificed using an intracardiac injection of pentobarbital. Then, the eyes were enucleated, placed in Davidson fixative medium and inserted in paraffin before sectioning. Histological studies were performed in all study eyes. Tissue slices of 10 um from the frozen sections were cut until the sclerotomy location was reached and were stained with hematoxylin and eosin. Digital photographs from histological slides were taken and studied using NIH Image software (version 1.62, National Institutes of Health, Bethesda, MD).

## Results

In our preliminary studies in the ten enucleated porcine eyes using 20 and 30 % pNIPAM, leakage through the sclerotomies occurred at a pressure of 10 mmHg of bottle height. With the higher concentration of 50 % pNIPAM, no leakage was observed until 80 mmHg of bottle height. The untreated wounds leaked at a pressure of 5 mmHg of bottle height, and the sutured wounds leaked at a pressure of 70 mmHg.

For the eight rabbits used in this study, no mechanical lesions apart from the application sites themselves were observed from the glue application. The liquid polymer spread out and stuck to the conjunctiva around the sclerotomy wound in three rabbits. In each of the eyes with scleral temperature of 32 °C or above that received pNIPAM, a smooth and shallow white halo corresponding to the pNIPAM glue appeared around the sclerotomy site immediately after injection. The size and shape of the polymerized glue continued unchanged during the follow up period in all of the eyes. In the two rabbits in which the temperature was below 32 °C, the pNIPAM glue was not able to polymerize and stick to the scleral tissue as expected.

**Fig. 2:**
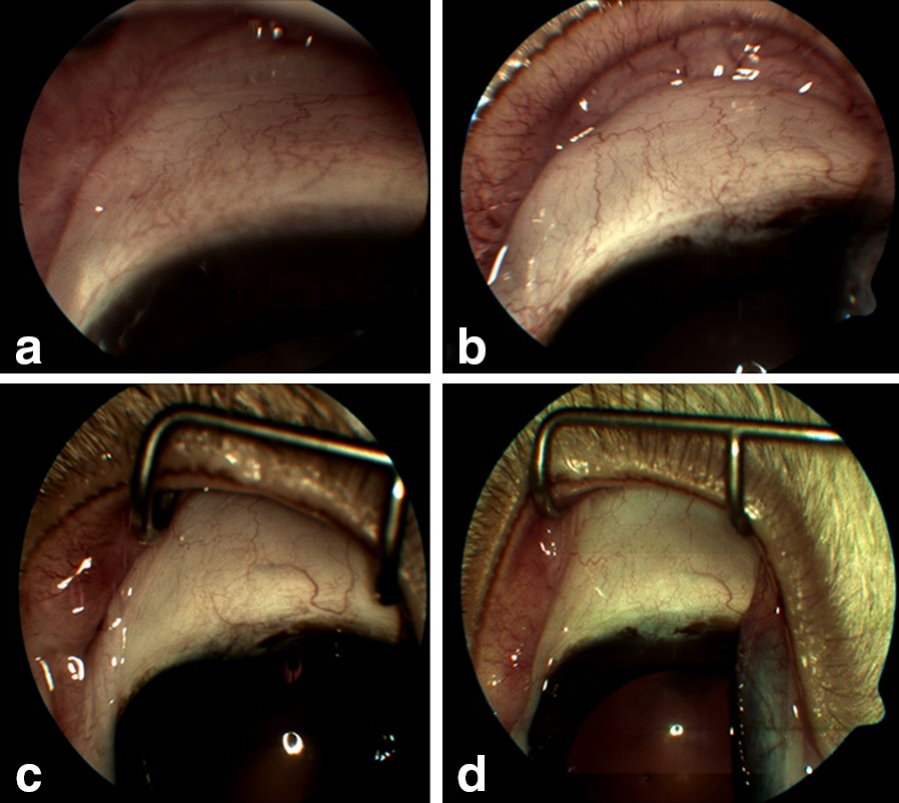
**a**–**d** External color photographs showing the appearance of the conjunctiva over the sclerotomy wound site 15 days (**a**), 1 month (**b**), 3 months (**c**), and 6 months (**d**) after the surgical procedure in one of the 8 study rabbits. The conjunctiva swelling decreased through the follow-up period

The efficacy of the scleral closure was evaluated by regular examinations. There was excellent wound closure of the scleral incisions both at the time of surgery and at the time of sacrifice. No animal showed any signs of wound dehiscence, infection, or cyst formation at any time. At 2 weeks, all incisions were well healed and difficult to detect via slit lamp biomicroscopy. No filtering bleb was noted indicative of leakage through the sclerotomies during the follow-up period. Similar results were observed through the six month period of the long-term chronic experiment (Fig. 2). Less inflammatory reaction occurred in the group of rabbits in which the scleral wound was closed with pNIPAM than in those rabbits in which vicryl sutures were used.

Except for small posterior subcapsular cataracts in two rabbits caused by accidental lens contact during the vitrectomy, no ocular abnormalities were noted in any of the eyes in the perioperative settings or during the follow-up period. Cornea, retina, and vitreous remained unaffected, and no abnormal inflammatory reaction or endophthalmitis was observed. Intraocular pressure remained normal, ranging from 11 to 18 mmHg (mean: 14 mmHg). The difference in intraocular pressure between the two eyes of each animal was always less than 4 mmHg at each examination.

**Fig. 3:**
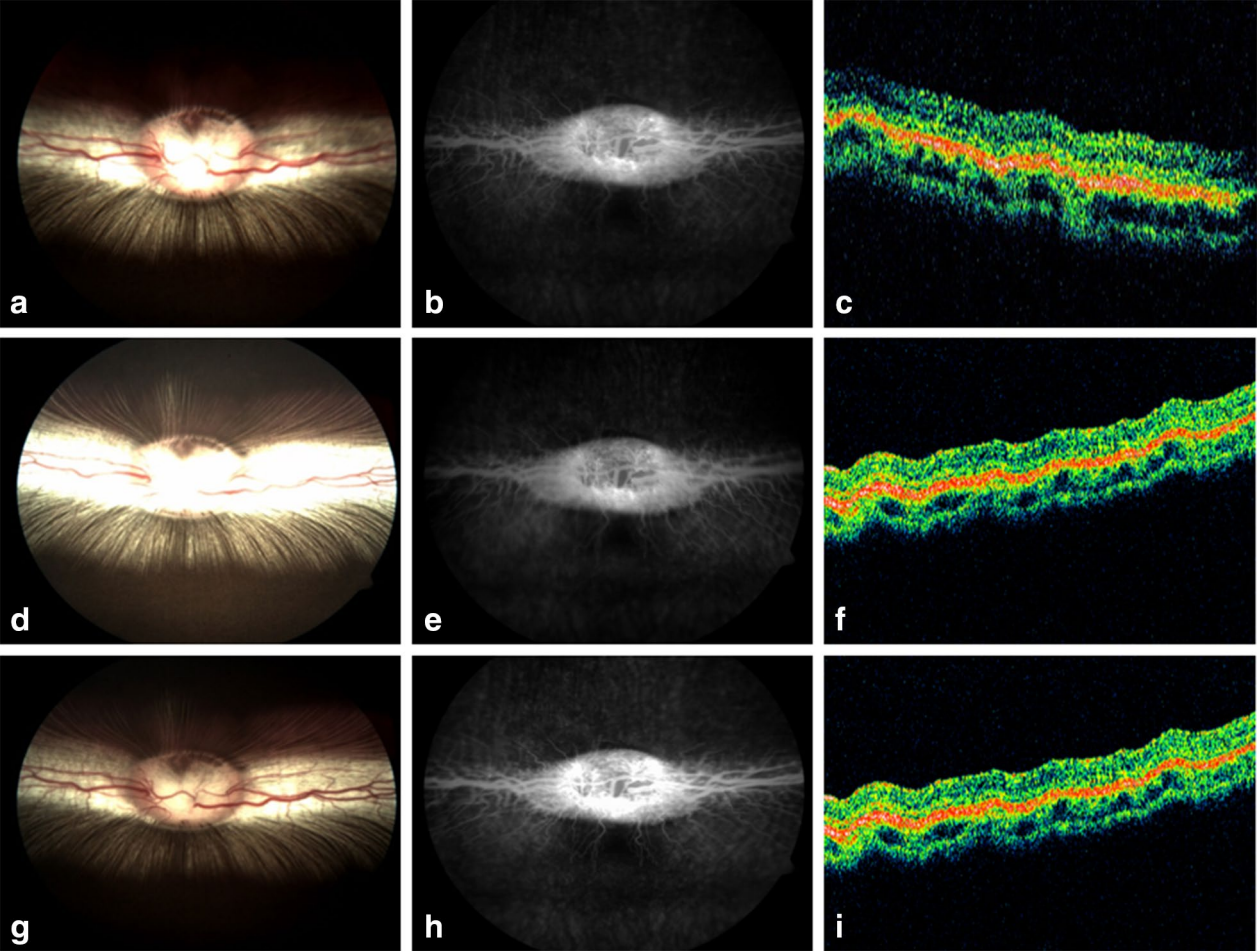
**a**–**i** Color fundus photographs, fluorescein angiography, and optical coherence tomography from one of the 8 rabbits at the baseline (**a**–**c**), 3 months (**d**–**f**), and 6 months (**g**–**i**) follow-up intervals. No retinal abnormalities were observed during the follow-up period

Fundus color photographs, OCT, and FA revealed no significant abnormalities during the follow-up period (Fig. 3). Throughout the course of the study, the mean b-wave amplitude ratios of ERG recordings (eyes with pNIPAM/left eyes) remained close to 1. Paired comparisons of the preoperative b-wave ratios versus the ratios at each time period showed no significant differences when evaluated with the Student *t* test.

**Fig. 4:**
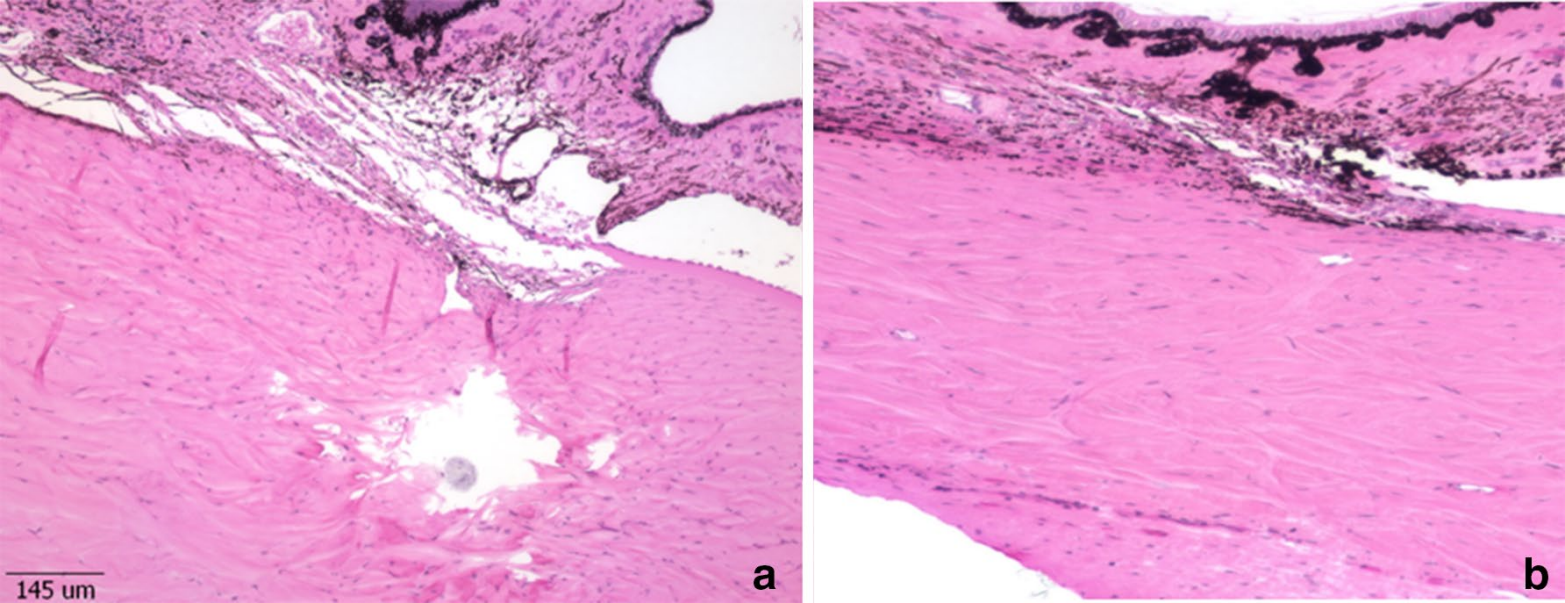
Light microscopy of the retina and sclera. The sclera tissue is facing downwards. **a** One month after PNIPAM application, a foreign body corresponding to PNIPAM material is observed. **b** Wound healing without any existence of adhesive material is observed 6 months after the PNIPAM application

In the histologic sections, at the sclerotomy sites of the 2 rabbits from the mid-term chronic study group (14 and 30 days follow-up), a foreign body corresponding to pNIPAM material was observed. Wound healing without any existence of glue and a smooth overlying conjuctiva were observed at the sclerotomy site in the long-term chronic group (6 months follow-up). No abnormal fibrous proliferation or vitreous incarceration was identified in the histologic sections (Fig. 4).

## Discussion

Currently, 20-gauge sclerotomy wounds are usually closed with sutures, while most 23-gauge and 25-gauge wounds are considered to be self-sealing. Closure with sutures increases the chance of causing changes in the curvature of the eye, possibly resulting in refractive problems and slowing postoperative recovery [23, 24]. Sometimes, “self-sealing” sclerotomies continue to leak, leading to complications such as ocular hypotony and endophthalmitis [25, 26].

The ideal adhesive for extras- or intraocular use should be nontoxic, biocompatible, tolerated by ocular tissue (i.e., smooth surface), and inexpensive. Previous reports showed that adhesives such as cyanoacrylate and fibrin glue were ineffective for use in sclerotomy closure [4–6] Although *N*-isopropylacrylamide monomer is toxic to neural tissue, after polymerization, the pNIPAM molecule is no longer toxic. It is commonly used, for example, in cell and tissue cultures for its reversible cell adhesion properties. Interestingly, pNIPAM has also been used to stop bleeding in experimental liver injuries and no toxicity has been reported. This thermoresponsive, “smart” polymer has been postulated to have other potential applications in ophthalmic surgery and drug delivery [27–31].

In our feasibility study, pNIPAM was used to close 20-gauge sclerotomy wounds in rabbit eyes. The pNIPAM liquid has a low viscosity and is of a white color. The best bond strength is achieved by using a thin film of the glue. The adhesive formed an immediate strong bond between the scleral edges in an easy and reproducible manner. During surgery, the adhesive polymerized within a few minutes and had the necessary strength for initiation and maintenance of scleral apposition. As the pNIPAM glue polymerizes quickly inside the needle before application, we use a syringe without a needle. Consequently, a precise glue application was somewhat difficult to achieve since the glue spread out beyond the sclera incision edges. Future studies can better address the question of application. One also must be careful to isolate the conjunctival wound edges because any stray adhesive can easily glue other ocular structures.

The local effects of pNIPAM on follow up examination were minimal. A slit-lamp examination of the glue application site showed only moderate inflammatory changes at 1 week and only mild conjunctival hyperemia by 2 weeks. No ocular inflammatory signals were noticed by 4 weeks. Thus, the adhesive seems to be well tolerated by rabbit tissues. The adhesive bond is also a flexible bandage, which covers the entire scleral wound. Theoretically, this should decrease the possibility of postoperative bacterial contamination of the vitreous cavity. Thus, it can reduce the chance of endophthalmitis after vitrectomy surgery. Another advantage of pNIPAM could be a reduction in the chances of postoperative astigmatism in comparison with scleral wounds closed by sutures.

In the acute setting, with 20-gauge sclerotomies, there were no relevant complications such as low intraocular pressure. During the follow-up period, complications such as endophthalmitis, retinal folds, and choroidal detachment were not observed. All rabbits underwent successful closure of 20-gauge sclerotomy wounds when scleral temperature was above 32 °C. The wound closure created by pNIPAM was sufficient in all cases to maintain tissue apposition throughout the experimental period, i.e. to 14 days, 1 month, and 6 months.

Although the present study addressed only the closure of perpendicular 20-gauge sclerotomies by PNIPAM, future research in this field may focus on the feasibility of PNIPAM for closing transconjunctival and oblique 23, 25 and 27-gauge sclerotomies performed in the current microincision vitrectomy surgery (MIVS). Since we showed the conjunctiva around the scleral incision was normal without any inflammatory reaction 1-month after PNIPAM application, it is probable that this polymer has the potential for closing transconjuctival MIVS sclerotomies.

## Conclusion

Our study demonstrated that pNIPAM can be attached from the scleral surface, merely by changing the temperature of the scleral tissue. Theoretically, this reversibility property may offer the capability to remove the pNIPAM-sclera adhesion whenever necessary solely by using a lower temperature BSS in infusion. pNIPAM with microfabrication and patterning may have the potential to be used as a nanotechnological system for sutureless sclerotomies closure. In addition, pNIPAM also can have other uses in drug-delivery and ocular procedures. In conclusion, although clinical studies are required to evaluate its utility in patients undergoing pars plana vitrectomy, pNIPAM when used at a proper temperature seems to have the potential to be used as a thermosensitive reversible adhesive for 20-gauge scleral wound closure.

## Abbreviations

ERG: Electroretinography
FA: Fluorescein angiography
LCST: Lower critical solution temperature
OCT: Optical coherence tomography
pNIPAM: Poly-*N*-isopropylacrylamide
